# Relationship Between Mastalgia And Anxiety-Depression: An Observational Study

**DOI:** 10.7759/cureus.12734

**Published:** 2021-01-16

**Authors:** Mehmet kağan Katar, Murat Başer

**Affiliations:** 1 General Surgery, Yozgat Bozok University Faculty of Medicine, Yozgat, TUR

**Keywords:** breast pain, mastodynia, mastalgia, anxiety, depression

## Abstract

Background: Mastalgia is one of the most common breast disorders and may adversely affect a person's daily activities and health-related quality of life, along with possible psychological discomfort. In our study, we investigated whether there is a relationship between mastalgia and anxiety and depression.

Methods: In this prospective study, patients with mastalgia comprised the mastalgia group (n=130) and those without any complaints were included as the control group (n=128). Sociodemographic characteristics such as age, marital status, and educational level were recorded. Both groups were evaluated using the Beck Anxiety Inventory (BAI) and the Beck Depression Inventory (BDI).

Results: The mean age of the participants was 34.45 ± 6.06 years for the mastalgia group and 35.15 ± 6.39 years for the control group. There was no statistically significant difference between the two groups in terms of age (p = 0.371), marital status (p = 0.336), job status (p = 0.320) or educational level (p = 0.285). However, the anxiety scale and depression scale scores were significantly higher in the mastalgia group compared to the control group (p < 0.001). In addition, the evaluation showed that the BAI and BDI scores were significantly high in the cyclic mastalgia group (p < 0.001). The correlation analyses of the patient group revealed that there was a positive correlation between duration of disease and BAI and BDI scores [(r=0.453, p<0.001); (r=0.228, p=0.009), respectively]. Similarly, there was a positive correlation between educational level and BAI and BDI scores [(r=0.579, p<0.001); (r=0.523, p<0.001), respectively].

Conclusion: In our study, anxiety and depression were found to be more common in mastalgia patients than healthy controls for various reasons. Thus, physicians should look for any signs of psychological discomfort in patients presenting with mastalgia and, if necessary, consult a psychiatrist.

## Introduction

Mastalgia is the most common breast disorder that affects women [1], and has been reported to have a prevalence of up to 52% [2]. Although many reasons for this have been implicated, including nutrition and psychological and hormonal factors, its etiology has not been elucidated [3].

In some studies, mastalgia has been reported to adversely affect the daily activities of women and to reduce their health-related quality of life [4]. In fact, in one study, mastalgia had negative effects on quality of life and also on sexual activity in 41%, sleep quality in 35%, and work-life in 5% [2]. Mastalgia can also create an additional psychological burden by affecting daily life so much. In addition, due to increasing public awareness of breast cancer, the concern that mastalgia may be a cancer symptom is increasing among women today. We anticipate that this anxiety may facilitate the occurrence of psychological discomfort in those suffering from mastalgia.

Considering all these factors, we think that the stress level may be high in patients suffering from mastalgia for various reasons, therefore anxiety and depression may be higher in these patients than in the normal population. In this study, we aim to evaluate whether there is a relationship between mastalgia and anxiety and depression.

## Materials and methods

This is a prospective study conducted in the Department of General Surgery, from May 2019 to July 2019. Ethical approval was obtained from the local Clinical Research Ethics Committee prior to the study being carried out according to the Helsinki Declaration. The study was conducted prospectively, and signed informed consent was obtained from all subjects.

Sample size

The sample size was based on the study of Yılmaz et al. who observed that Generalized Anxiety Disorder-7 (GAD-7) test score in cases was 8.06±3.8 as compared to 3.96±2.7 in controls [5]. Taking these values as reference, the minimum required sample size with 99% power of study and 1% level of significance is 30 patients in each study group. To reduce the margin of error and increase the strength of the study, all patients who admitted to the general surgery clinic due to mastalgia between the dates determined before the study and met the inclusion and exclusion criteria were included in the study.

Women aged 18-55 years who were admitted to our outpatient clinic with mastalgia were included in the study. Patients with a previous diagnosis of breast cancer, direct trauma to the breast in the last month, any invasive procedure (surgical intervention, biopsy, etc.) to the breast, pregnancy, breastfeeding, mastalgia for less than one year, hormonal contraception of hormone replacement therapy use, an active psychiatric disease or psychiatric drug use, any sign of infection in the breast, palpable breast mass on physical examination, benign breast disease that could cause mastalgia and cystic or solid lesions larger than 10 mm in the breast were excluded from the study.

**Figure 1:**
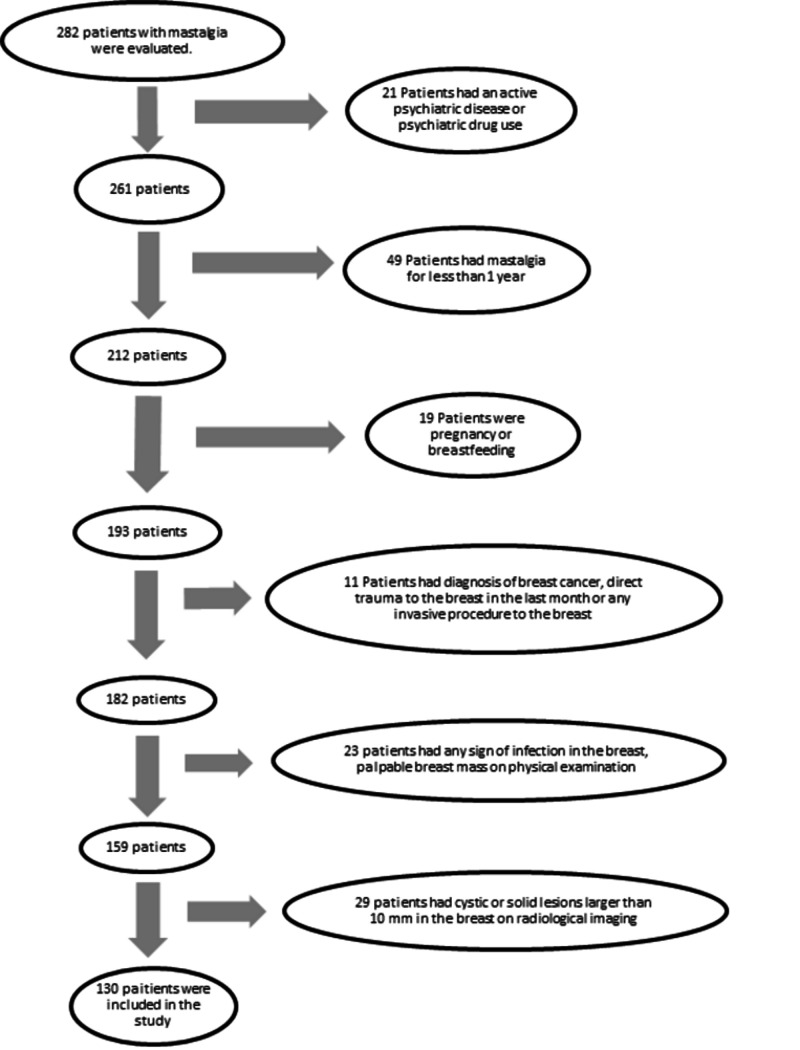
Study flow chart

A total of 130 patients who met the inclusion and exclusion criteria, volunteered to participate in the study and complained of mastalgia were placed in the mastalgia group. A flow chart of the study population is admitted in Figure 1. The control group consisted of medical personnel working in the same hospital and their relatives who had similar demographic characteristics to the mastalgia group, no complaints, no diagnosis of any psychiatric illness and no history of using any psychiatric drugs. The same exclusion criteria were used for both groups. Finally, 128 healthy women who met the inclusion criteria and volunteered to participate were included in the study.

Sociodemographic characteristics such as age, marital status, and educational level were recorded. Moreover, the age at the start of mastalgia and the relation between mastalgia and menstrual cycle were also evaluated [6]. All women participating in the study were evaluated using the Beck Anxiety Inventory (BAI) and the Beck Depression Inventory (BDI).

The BAI aimed to determine the frequency of anxiety symptoms. It was developed by Beck et al. (1988) and is used as a Likert-type self-assessment scale [7]. It consists of 21 items and each item is scored between 0 and 3. The higher the total score, the higher the anxiety experienced by the person.

The BDI, also developed by Beck et al., is a self-assessment scale that includes 21 symptom categories [8]. The purpose of the scale is not to diagnose depression but to objectively digitize the degree of symptoms. It measures the physical, emotional and cognitive symptoms seen in depression. The highest score achievable is 63 and a high score indicates more severe depression.

Statistical analysis

The Statistical Package for the Social Sciences (SPSS 22.0, IBM Inc., Chicago, IL, USA) program was used for statistical analysis of the data. The Kolmogorov-Smirnov test was performed to assess the distribution of normality and the chi-square test to compare groups with regard to categorical variables. Student‘s t-test was applied to compare two groups of data showing a normal distribution and the Mann-Whitney U test was performed for data not showing a normal distribution. Pearson’s correlation test was used for examination of normally distributed data and Spearman’s correlation test for data that were not dispersed normally. A p value of < 0.05 was considered to be statistically significant.

## Results

There was no significant difference between the mastalgia group and the control group in terms of age, education level, marital status, and job status. The sociodemographic characteristics of the groups are shown in Table 1.

**Table 1:** The sociodemographic characteristics

	Mastalgia Group n=130	Control Group n=128	p value
Age^1^	34.45±6.06	35.15±6.39	0.371^a^
Educational Level^1^	10.15±3.32	9.90±3.42	0.618^b^
Marial status			0.336^c^
Single (%)	26 (20)	32 (25)	
Married (%)	104 (80)	96 (75)	
Job Status			0.320^c^
Employed (%)	48 (36.9)	58 (45.3)	
Unemployed (%)	71 (54.6)	58 (45.3)	
Student (%)	11 (8.5)	12 (9.4)	
^1^Data were shown as mean ± standard deviation. ^a^p-values were calculated by the independent t-test. ^b^p-values were calculated by Mann-Whitney test. ^c^p-values were calculated by the chi-square test.			

The mean duration of disease in the mastalgia group was 2.04±1.11 years and the mean age of disease onset was 32.42±5.74 years. Both cyclic and non-cyclic mastalgia was found, in 60.7% (n = 79) and 39.3% (n=51), respectively. Evaluation showed that the BAI and BDI scores were significantly high in the cyclic mastalgia group (p<0.001).

Mean BAI score for the mastalgia group being significantly higher than that for the control group (p<0.001). Similarly, the mean BDI scores for the mastalgia group being significantly higher than that for the control group (p<0.001). BAI and BDI results and statistical comparisons are shown in Table 2.

**Table 2:** BAI and BDI results and statistical comparisons

	Mastalgia Group	Control Group	p value^*^
BAI score	14 (0-44)	6 (0-31)	< 0.001
BDI score	10 (0-24)	5 (0-30)	< 0.001
BAI: Beck Anxiety Inventory. BDI: Beck Depression Inventory. ^*^p-values were calculated by Mann-Whitney test			

**Figure 2:**
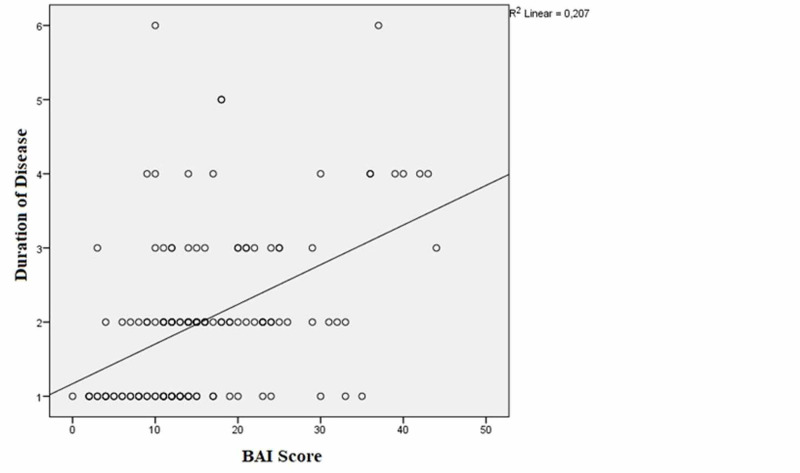
The correlation between duration of the disease and the Beck Anxiety Inventory (BAI) scores

**Figure 3:**
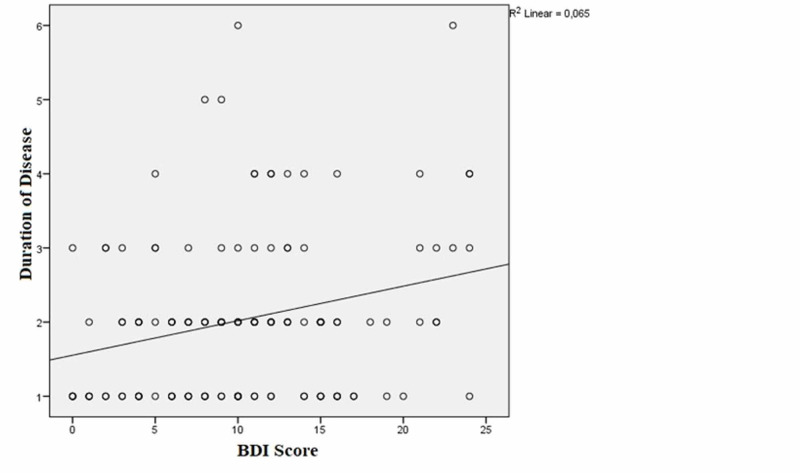
The correlation between duration of the disease and the Beck Depression Inventory (BDI) scores

The correlation analyses of the patient group revealed that there was a positive correlation between duration of disease and BAI and BDI scores [(r=0.453, p<0.001); (r=0.228, p=0.009), respectively] (Figures 2-3).

**Figure 4:**
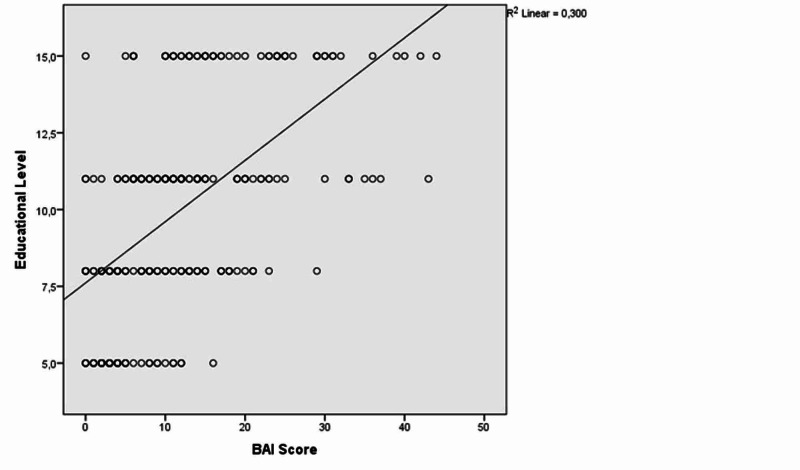
The correlation between educational level and Beck Anxiety Inventory (BAI) scores

**Figure 5:**
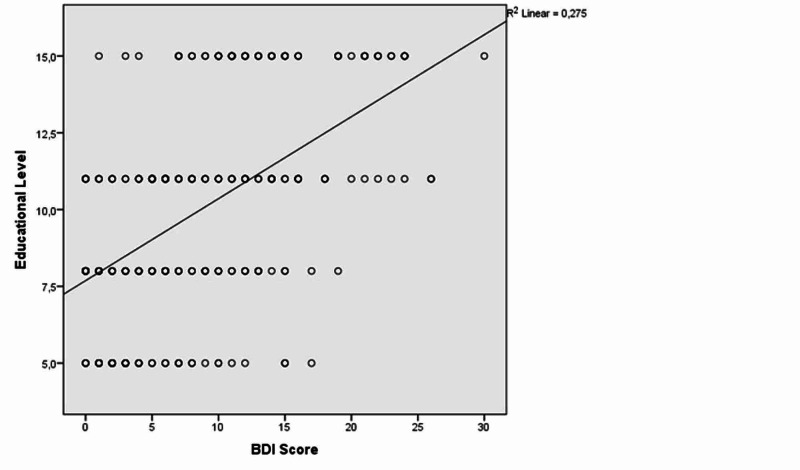
The correlation between educational level and Beck Depression Inventory (BDI) scores

Similarly, there was a positive correlation between educational level and BAI and BDI scores [(r=0.579, p<0.001); (r=0.523, p<0.001), respectively] (Figures 4-5).

## Discussion

In this study, the BAI score was found to be significantly higher in the mastalgia group compared to healthy controls. This result is in parallel with some studies in the literature [9-10]. In a study conducted by Balci et al., it was reported that the BAI score in the mastalgia group was significantly higher than in the healthy control group but the fact that patients were included in the mastalgia group without any examination or imaging methods to investigate the etiology of mastalgia limits the study [11]. However, in our study, patients were examined in detail by a physician, and any necessary imaging methods were applied before they were included in the mastalgia group, meaning that only those with no organic pathology were included in the study.

Also in our study, it was determined that the BDI score in the mastalgia group was significantly higher than in healthy controls. However, there is conflicting information in the literature on this subject. For example, Colegrave et al. reported that depression in the mastalgia group was significantly higher than in healthy controls [10] and also Ramirez et al. showed that depression is more common in those suffering from severe mastalgia than non-severe mastalgia [12]. In contrast, Kanat et al. compared 40 premenopausal mastalgia patients with 40 healthy controls with similar demographic characteristics and found no statistically significant difference despite the high level of depression in mastalgia patients [13]. Similarly, Öztürk et al. reported no significant difference in the depression score between healthy controls and those with mastalgia symptoms [14]. We believe that larger multicentre case-series studies are needed to elucidate this issue.

There may be various reasons for the high levels of anxiety and depression in the mastalgia group. One of the main reasons may be the increasing awareness of breast cancer in society in recent years, along with the increase in imaging methods, that has led to an increase in the prevalence of breast cancer. Although breast cancer occurs in only 0.4% of women presenting with mastalgia, it has been reported in some studies that this may raise the concern that mastalgia may be one of the symptoms of malignancy in women [3-15]. A study supporting this concern was conducted by Plu-Bureau et al. [16] reporting a significant increase in the risk of breast cancer in patients with benign breast pathology together with mastalgia. In other words, worrying about breast cancer may be one of the causes of anxiety disorder and depression.

Another reason is that mastalgia negatively affects one's daily life activities and has been reported to adversely affect health-related quality of life due to this negative effect [13]. The impact of daily living activities and the consequent decline in health-related quality of life may lead to a more stressful lifestyle. Indeed, a study that supports this idea was carried out by Eren et al. [17] In this study, which included 700 women, 500 of whom were mastalgia patients, the number of patients who identified themselves as having a “stressful lifestyle” was significantly higher in the mastalgia group. Again, psychological stress is involved in the pathophysiology of depressive disorder in the literature [18].

In our study, the BAI and BDI scores were found to be significantly high in cyclic compared with non-cyclic mastalgia patients. Although speculative, this may be explained by the woman’s expectation of cyclic symptoms induced by cyclic hormonal changes, which seems a rational reason for anxiety.

Another point that we want to emphasize is that if anxiety or depression intensifies, it may become increasingly difficult to control mastalgia. In one study, anxiety and depression were reported to be influential in the severity and duration of mastalgia and a vicious circle can occur [19]. This means that, after development, when mastalgia is not controlled it may cause anxiety or depression. With the development of anxiety or depression, the severity of mastalgia may increase or the duration of the disease may be prolonged. This may make it difficult to treat both mastalgia and anxiety and depression. To prevent or break this vicious circle, it would be appropriate to prevent the formation of anxiety and depression or to include psychological and emotional support from the outset in the treatment protocol. The importance of psychological evaluation, as well as a good physical examination, is thus revealed in mastalgia patients.

Furthermore, a positive correlation was found between educational level and the BAI and BDI scores in our study. This result supports our hypothesis: that the awareness of breast cancer may also increase with an increase in educational level. In other words, the worry that mastalgia may be a leading symptom for breast cancer may be more common in women with higher levels of education. Thus, this group of women may have more anxiety and depression. However, this information should be supported by further studies. Moreover, we found a positive correlation between prolongation of disease duration and the BAI and BDI scores. Accordingly, when mastalgia is not controlled and the disease duration is prolonged, the risk of anxiety and depression increases. This result indicates that if mastalgia is detected, the necessary intervention should be performed as soon as possible.

Some factors limit our study, including the relatively limited number of patients, the fact that the study was conducted in one center, that the scale used to investigate anxiety and depression was filled out by the patient, and that it had no diagnostic features. However, despite all the limitations, we think that the fact that our study was conducted in the productivity age and young-adult age group and that there was no organic pathology in any of the patients included in the study increased the strength of our study. As a result of our study, we believe there will be awareness among physicians that mastalgia should not be underestimated, especially in that age group.

Mastalgia is one of the most common breast disorders but it is not possible to classify the symptom with a scientific scale as it is a subjective complaint stated by the patient. Therefore patient groups and healthy groups are formed according to this subjective statement. However, some definitive factors like the patient’s pain threshold or mood may possibly influence the groups. Keeping this in mind, we have to find an answer to the conflict: Which is more rationale? Do women with mastalgia have high anxiety or do women with high anxiety levels complain more about mastalgia? We have to study with psychiatrists more in order to explain the relationship between mastalgia and anxiety.

## Conclusions

It should be kept in mind that mastalgia can cause serious psychological disorders, including anxiety and depression, as well as seriously affecting the health-related quality of life of women. Furthermore, the physician should be vigilant in terms of psychological disorders in patients presenting with this complaint and should seek psychiatric consultation when necessary. Thus, we want to emphasize that the treatment of mastalgia will improve with the necessary intervention for those with anxiety and depression.

## References

[1] Suhani S, Srivastava A, Parshad R, Seenu V, Kataria K, Rathode Y (2019). Complete estrogen blockade in recalcitrant mastalgia: a pilot study. Indian J Surg.

[2] Scurr J, Hedger W, Morris P, Brown N (2014). The prevalence, severity, and impact of breast pain in the general population. Breast J.

[3] Kataria K, Dhar A, Srivastava A, Kumar S, Goyal A (2014). A systematic review of current understanding and management of mastalgia. Indian J Surg.

[4] Santen RJ, Mansel R (2005). Benign breast disorders. N Engl J Med.

[5] Yılmaz EM, Çelik S, Arslan H, Değer D (2015). Relation between mastalgia and anxiety in a region with high frequency of posttraumatic stress disorder. J Breast Health.

[6] Smith RL, Pruthi S, Fitzpatrick LA (2004). Evaluation and management of breast pain. Mayo Clin Proc.

[7] Beck AT, Epstein N, Brown G, Steer RA (1988). An inventory for measuring clinical anxiety: psychometric properties. J Consult Clin Psychol.

[8] Beck AT, Ward CH, Mendelson M, Mock J, Erbaugh J (1961). Beck depression inventory (BDI). Arch Gen Psychiatry.

[9] Joyce D, Alamiri J, Lowery AJ, Downey E, Ahmed A, McLaughlin R, Hill ADK (2014). Breast clinic referrals: can mastalgia be managed in primary care?. Ir J Med Sc.

[10] Colegrave S, Holcombe C, Salmon P (2001). Psychological characteristics of women presenting with breast pain. J Psychosom Res.

[11] Balcı N, Kantekin V, Sunay D (2013). Mastalgia, anxiety and related factors: case-control study. Turk J Fam Pract.

[12] Ramirez AJ, Jarrett SR, Hamed H, Smith P, Fentiman IS (1995). Psychosocial adjustment of women with mastalgia. Breast.

[13] Kanat BH, Atmaca M, Girgin M (2016). Effects of mastalgia in young women on quality of life, depression, and anxiety levels. Indian J Surg.

[14] Ozturk AB, Tugal O, Ozenli Y (2015). Somatization in mastalgia patients: is there a relationship between mastalgia and somatization symptoms?. Sch J App Med Sci.

[15] Duijm LEM, Guit GL, Hendriks JHCL, Zaat JOM, Mali WPTM (1998). Value of breast imaging in women with painful breasts: observational follow up study. BMJ.

[16] Plu-Bureau G, Le MG, Sitruk-Ware R, Thalabard JC (2006). Cyclical mastalgia and breast cancer risk: results of a French cohort study. Cancer Epidemiol Biomarkers Prev.

[17] Eren T, Aslan A, Ozemir IA, Baysal H, Sagiroglu J, Ekinci O, Alimoglu O (2016). Factors effecting mastalgia. Breast Care.

[18] McEwen BS (2008). Central effects of stress hormones in health and disease: Understanding the protective and damaging effects of stress and stress mediators. Eur J Pharmacol.

[19] Mirghafourvand M, Ahmadpour P, Rahi P (2016). Relationship between depression and anxiety with the severity and length of cyclic mastalgia in women. Iran J Obstet Gynecol Infertil.

